# Long-Term Efficacy and Safety of Modified Canaloplasty Versus Trabeculectomy in Open-Angle Glaucoma

**DOI:** 10.3390/life13020516

**Published:** 2023-02-13

**Authors:** Julia V. Stingl, Felix M. Wagner, Sarah Liebezeit, Raphael Baumgartner, Helene Spät, Alexander K. Schuster, Verena Prokosch, Franz Grehn, Esther M. Hoffmann

**Affiliations:** 1 Department of Ophthalmology, University Medical Center of the Johannes Gutenberg-University Mainz, 55131 Mainz, Germany; 2 Regiomed Clinic Masserberg, 98666 Masserberg, Germany; 3 Department of Anaesthesiology, Hospital Worms, 67550 Worms, Germany; 4 Department of Ophthalmology, University of Cologne, 50937 Cologne, Germany

**Keywords:** trabeculectomy, canaloplasty, glaucoma surgery, open-angle glaucoma, long-term outcome

## Abstract

Background: To evaluate the long-term efficacy and safety of modified canaloplasty versus trabeculectomy in open-angle glaucoma. Methods: In total, 210 subjects with open-angle glaucoma were included. 70 were treated with Mitomycin C-augmented modified canaloplasty with enhanced subconjunctival filtration and 140 with Mitomycin C-augmented trabeculectomy. Cases were matched 1:2 by sex and age. Results: In canaloplasty and trabeculectomy groups, 61.4% and 57.9% of participants were female. Mean age was 60.0 ± 13.9 and 63.0 ± 12.2 years, median follow-up time was 4.6 [IQR 4.3, 5.05] years and 5.8 [IQR 5.4, 6.3]. Strict success was achieved in 20.0% and 56.4%, complete success in 24.3% and 66.4%, and qualified success in 34.3% and 73.6% (each *p* < 0.001). Kaplan–Meier survival analysis showed a better survival probability for trabeculectomy than for canaloplasty (*p* < 0.001) and Cox regression analysis revealed an HR of 6.03 (95%-CI 3.66, 9.93, *p* < 0.001) after canaloplasty. Trabeculectomy showed superiority in terms of IOP decrease (9.2 ± 7.9 mmHg vs. 13.7 ± 10.4 mmHg, *p* = 0.002), use of AGM (50.0% vs. 10.7%, *p* < 0.001), and the number of revision surgeries (41.4% vs. 21.4%, *p* = 0.004). Occurrence of complications was similar in both groups (14.5% vs. 7.5%, *p* = 0.19). Conclusions: Trabeculectomy showed superiority in efficacy and equality in safety compared to modified canaloplasty.

## 1. Introduction

Glaucoma affects 3.54% of people aged 40 to 80 years worldwide [1] and is the second leading cause of irreversible blindness [2] due to the loss of retinal ganglion cells. Although glaucoma is a multifactorial disease, most treatment strategies target intraocular pressure (IOP) reduction [3]. If IOP control is insufficient despite the application of topical antiglaucomatous medication (AGM), or if AGM intolerance occurs, a surgical approach is necessary to prevent further progress of ganglion cell loss.

Today, trabeculectomy is the standard procedure when it comes to glaucoma surgery [4]. Outcomes have markedly improved since its introduction roughly 50 years ago [5,6], among others due to applications of antifibrotics such as mitomycin C (MMC) and 5-fluorouracil (5-FU) to prevent postoperative bleb fibrosis [7,8]. However, these agents have been attributed to the occurrence of severe adverse events such as hypotony, bleb leak, choroidal detachment or hemorrhage, and endophthalmitis [4,9]. These complications are particularly feared in highly myopic eyes, but also short axial length bears risks such as the development of malignant glaucoma. To prevent these adverse events, so-called nonpenetrating procedures comprising deep sclerectomy, viscocanalostomy, and canaloplasty have been developed [10]. The efficacy and success of nonpenetrating surgeries have been discussed in the literature. A Cochrane review of randomized controlled trials found similar odds for success after deep sclerectomy and trabeculectomy, whereas the odds for success after viscocanalostomy amounted to only a third of the odds for success after trabeculectomy [11]. Another systematic review reported a higher efficacy of trabeculectomy in reducing IOP with a mean difference of −1.53 mmHg compared to deep sclerectomy, −3.84 mmHg compared to viscocanalostomy and −4.40 mmHg compared to canaloplasty 12 months after surgery [10]. They further found that enhancement by Mitomycin C lowered the mean difference of IOP between trabeculectomy and deep slerectomy from −2.65 to −0.83 mmHg after 6 months [10], indicating a better performance of nonpenetrating procedures when applying antifibrotic substances.

Most studies concerning this issue only offer a limited follow-up time span [12]. The aim of this study was thus to compare the success, efficacy, and safety of MMC-augmented modified canaloplasty and MMC-augmented trabeculectomy after a minimum follow-up time of 3 years.

## 2. Materials and Methods

### 2.1. Patient Cohort

This retrospective cohort study was conducted at the Department of Ophthalmology of the University Medical Center Mainz, Germany. Patients with open-angle glaucoma, who had undergone a modified canaloplasty between February 2016 and January 2018 were enrolled in this study. An electronic surgical case register was searched, and the subjects were manually reviewed for eligibility. Inclusion criteria were age 18 years or older and a diagnosis of open-angle glaucoma without prior filtering surgery. We identified 133 eligible patients with a minimum follow-up of three years after surgery. In total, 23 patients were excluded because the surgery did not comply with the standard surgery procedure protocol. Thirty patients were lost to follow-up (i.e., have not been examined by the Department of Ophthalmology Mainz or by the local ophthalmologist after surgery and were not reachable by phone) and thus excluded. Of 10 patients, both eyes were treated with modified canaloplasty and were eligible for inclusion; however, only the right eyes were included to facilitate the data evaluation. The remaining 70 cases were matched by sex and age with two trabeculectomy cases each (n = 140). Trabeculectomy cases were treated between January 2013 and February 2017 at the same center. The requirement for informed consent was waived by the ethics committee of the medical board of Rhineland-Palatinate according to regional laws. The study adhered to the tenets of the Declaration of Helsinki.

Collected data were fully pseudonymized and comprised demographics, glaucoma diagnosis, maximum preoperative IOP and pre- and postoperative IOP, and number of AGM and revision surgeries. The diagnosis was graded into three groups: primary open-angle glaucoma (comprising primary open-angle glaucoma, ocular hypertension, and juvenile open-angle glaucoma), secondary open-angle glaucoma (comprising pseudoexfoliative glaucoma, pigmentary glaucoma, other secondary glaucoma) and normal-tension glaucoma. If the necessary data were not available in the medical records, information was obtained from either patients or treating ophthalmologists.

### 2.2. Surgery

Surgeries were conducted either in topical, retrobulbar, or general anesthesia. Indications for surgery were uncontrolled IOP exceeding the individual target pressure which was between 10 and 18 mmHg and lower in the case of a more severe glaucomatous optic disc damage, faster rate of progression, lower untreated maximum IOP, longer life expectance, thinner central corneal thickness, positive family history of glaucoma, the status of the fellow eye and in light of patients’ preferences [13]. Surgery was recommended to the patients if AGM was not sufficient for IOP control or if the progression of nerve fiber loss (detected either by the deterioration of visual fields or thinning of peripapillary retinal nerve fiber layer in OCT examination) occurred despite exhausted local therapy or if the eye drops were not tolerated. In the case of ocular hypertension, surgery was only conducted if AGM was not sufficient to decrease IOP below 25 mmHg or if eye drops were not tolerated and the patient actively sought a surgical solution after discussion of glaucoma risk.

#### 2.2.1. Modified Canaloplasty

For modified canaloplasty, a fornix-based conjunctival flap was dissected. As shown in a previous meta-analysis, IOP decrease can be enhanced by the application of antifibrotic substances. Thus, a sponge soaked with MMC (0.2 mg/mL) was placed below the conjunctiva for 3 min, followed by rinsing with 30 mL saline solution. A superficial 4 × 4 mm scleral flap was prepared followed by dissection of a second deep scleral flap of size 1.5 × 3 mm leaving only a thin scleral layer over the choroid to allow some filtration into the suprachoroidal space, reaching into the clear cornea. The roof of SC was carefully detached, and the inner scleral flap was removed. Then, the iTrack microcatheter was inserted over 360 degrees guided by illumination and injecting viscoelastic every 2 clock hours into SC. A 10–0 Polypropylene suture was tied to the distal tip of the catheter after reemerging from the opposite opening of SC. Then the microcatheter was withdrawn from the canal and the suture was threaded through the canal. To dilatate SC, the Polypropylene suture was tautened to give a moderate pressure to its inner wall. The scleral flap then was closed loosely with 2 to 4 sutures (at the discretion of the surgeon) to allow gentle filtration and the conjunctiva was sutured in a meander-like fashion.

Using this modified method of canaloplasty a three-way outflow is targeted: First, by dilatation and spanning of SC; second, by augmentation of suprachoroidal outflow by dissecting a deep scleral flap; and third, by gentle filtering function promoted by the use of MMC and loose scleral flap reattachment. Thereby, MMC is used to prevent both the subconjunctival space and the scleral flap from scarring with the aim of maintaining gentle filtration without targeting a bleb, which usually only forms with steady filtration.

#### 2.2.2. Trabeculectomy

A fornix-based flap was dissected followed by mobilization of Tenon’s capsule. A sponge soaked with MMC (0.2 mg/mL) was inserted under the conjunctiva for 3 min, followed by intensive rinsing with 30 mL saline solution. A 4 × 4 mm scleral flap of one-third scleral thickness was created, and a temporal paracentesis was made. An anteriorly placed trabeculectomy was conducted and a peripheral iridectomy was created. The scleral flap was closed with four 10–0 nylon sutures, two at the edges and two at the sides. The conjunctiva was closed with meander-shaped sutures described by Pfeiffer and Grehn [14]. To re-check the bleb and tightness of the sutures the anterior chamber was inflated with a balanced salt solution.

Following both procedures, 4 mg dexamethasone was injected under the conjunctiva of the inferior fornix.

### 2.3. Perioperative Management

Perioperative management was similar for both surgical procedures.

Antiglaucomatous medication (AGM) was stopped 2 to 4 weeks prior to the surgery and topical dexamethasone 4 times daily was prescribed for 5 days prior to surgery to reduce conjunctival injections to prevent scarring. IOP spikes were treated with oral acetazolamide. After surgery, antibiotic prophylaxis with ofloxacin was applied for 7 days and topical dexamethasone was prescribed 6 times daily tapering off over 6 weeks. During the in-patient stay, subconjunctival injections of 5 fluorouracil, suture lysis, and bleb massage were performed at the discretion of the surgeon.

### 2.4. Outcome Measures

Outcome variables followed the Guidelines on Design and Reporting of Glaucoma Surgical Trials [15]. The primary outcome variable was the proportion of surgical success. Secondary outcome variables were IOP, number of AGM, number of revision surgeries, surgical complications, and visual acuity.

To ensure comparability with previous studies [12,16,17,18], surgical success was differentiated into three categories:

- Strict success was defined as IOP reduction of at least 20% compared to preoperative IOP, IOP at follow-up between 5 and 18 mmHg, no use of AGM, and no revision surgery (target IOP according to the European Glaucoma Society Terminology and Guidelines for Glaucoma [13]).
- Complete success was defined as IOP at follow-up between 5 and 18 mmHg, no use of AGM, and no revision surgery.
- Qualified success was defined as IOP at follow-up between 5 and 18 mmHg under the use of AGM and no revision surgery.

Failure was further considered if IOP was <5 (with signs of hypotony maculopathy) or >18 mmHg, or if revision surgery was necessary.

Revision surgery was considered necessary when the IOP was above the individual target IOP and if topical therapy was not desired or tolerated by the patient or if topical therapy was not sufficient. Laser suture lysis or goniopuncture were not regarded as revision surgery.

### 2.5. Statistical Analysis

Descriptive statistics such as demographic and ocular characteristics were calculated as mean ± standard deviation for normally distributed continuous variables and median and interquartile range for nonnormally distributed variables. Categorial variables were described in absolute and relative frequencies.

Comparison of continuous variables was conducted with a Student’s *t*-test or a paired *t*-test for within-group comparisons, and with χ^2^ test for categorial variables. Survival analysis of complete surgical success was performed using Kaplan–Meier curves and Cox regression analysis. Tests for proportional hazard assumptions were conducted and Schoenfeld residues were calculated.

Statistical analysis was conducted with R (v4.1.3).

## 3. Results

Seventy eyes of 70 patients treated with modified canaloplasty (61.4% female), and 140 eyes of 140 patients treated with trabeculectomy (57.9% female) were included. The mean age was 60.0 ± 13.9 and 63.0 ± 12.2 years. The distribution of diagnosis differed slightly between the groups (*p* = 0.07): for example, 31.4% of the canaloplasty group consisted of secondary glaucoma whereas the proportion in the TE group was lower (18.0%). Reported maximum IOP was higher in the modified canaloplasty group (35.3 ± 11.5 mmHg) than in the trabeculectomy group (31.3 ± 10.6 mmHg, *p* = 0.02); however, measured preoperative IOP did not significantly differ between the two groups (24.0 ± 6.9 versus 25.3 ± 9.9 mmHg, *p* = 0.36). The duration of glaucoma at the time of surgery was similar in both groups (8.2 vs. 7.6 years, *p* = 0.36) and the mean defect of visual field examinations did not differ significantly (6.6 vs. 7.0 dB, *p* = 0.68). Baseline characteristics are shown in Table 1.

### 3.1. Surgical Success

Strict surgical success was achieved in 14/70 subjects (20.0%) for modified canaloplasty and in 79/140 (56.4%) subjects for trabeculectomy after a median follow-up time of 4.6 [IQR 4.3, 5.05] years for modified canaloplasty and 5.8 [IQR 5.4, 6.3] for trabeculectomy (*p* < 0.001). Complete success was attained in 17/70 (24.3%) cases versus 93/140 (66.4%) cases (*p* < 0.001) and qualified success was seen in 24/70 (34.3%) cases versus 103/140 (73.6%) cases (*p* < 0.001).

**Figure 1:**
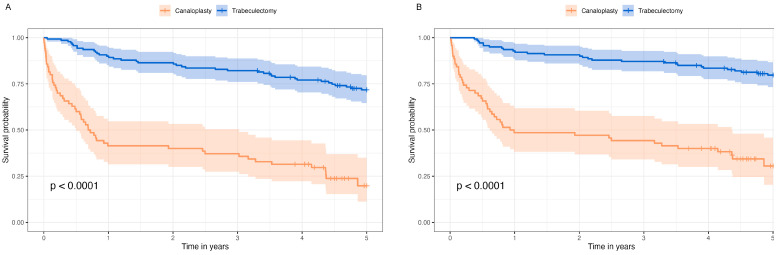
Kaplan–Meier survival analysis of complete surgical success (**A**) and qualified surgical success (**B**) after modified canaloplasty and trabeculectomy.

Figure 1A shows the survival probability of complete surgical success and Figure 1B the survival probability of qualified surgical success in Kaplan–Meier survival analysis. The median survival probability of modified canaloplasty was 0.75 years [95%-CI 0.56, 2.50] for complete success and 0.96 years [95%-CI 0.62, 4.36] for qualified success.

Cox regression analysis of qualified success showed a hazard ratio (HR) of 6.03 for modified canaloplasty (*p* < 0.001). Survival probability was independent of sex, age, and diagnosis (Table 2) and also from preoperative visual field MD and duration of glaucoma (Supplemental Material File S1). Another Cox regression analysis including only data from the first year revealed an HR of 11.20 (95%-CI 5.3, 23.5; *p* < 0.001) for modified canaloplasty, whereas the Cox regression of years 1 to 5 showed an HR of 2.48 (95%-CI 1.1, 5.6; *p* = 0.03). However, the test for proportional hazard assumption was statistically significant (global tests *p* = 0.02) and thus not fulfilled.

### 3.2. Intraocular Pressure

**Figure 2:**
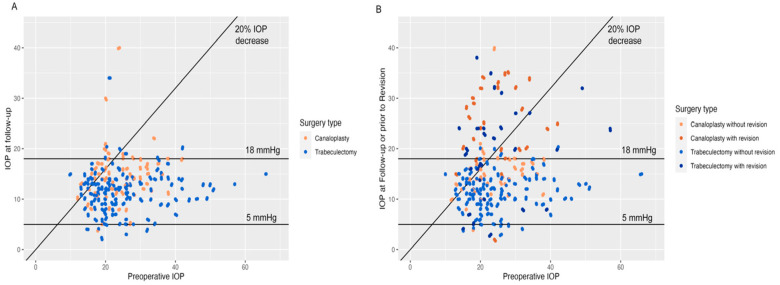
Intraocular pressure development. (**A**): Intraocular pressure before surgery and at last follow-up in both groups. (**B**): Intraocular pressure before surgery and either at the last follow-up (light colors) or prior to revision surgery (dark colors), if applicable. Abbreviations: IOP, intraocular pressure.

Preoperative IOP was 24.0 ± 6.9 mmHg for modified canaloplasty cases and 25.3 ± 9.9 mmHg for trabeculectomy cases (*p* = 0.35). IOP at follow-up amounted to 14.5 ± 5.2 mmHg and 11.6 ± 4.1 mmHg (*p* < 0.001), respectively. The mean percentage IOP decrease at the last follow-up examination after 4.6 and 5.8 years was 36.0 ± 28.0% and 48.3 ± 25.9% (*p* = 0.002), and absolute IOP decrease was 9.2 ± 7.9 mmHg versus 13.7 ± 10.4 mmHg (*p* = 0.002). Figure 2A shows a scatterplot of preoperative IOP and IOP at the last follow-up examination.

When cases without AGM and revision surgery were included to rule out any influence of postoperative procedures, i.e., all failures by AGM or revision surgery were excluded, the mean IOP at the last follow-up was 13.1 ± 3.3 mmHg after modified canaloplasty compared to 11.3 ± 3.6 mmHg after trabeculectomy (*p* = 0.03) with significant difference of absolute and relative IOP decrease (9.6 ± 7.3 mmHg versus 14.2 ± 9.9 mmHg, *p* = 0.048 and 38.0 ± 20.4% versus 50.6 ± 24.0%, *p* = 0.03). 

If—in case a revision surgery was conducted—IOP prior to the first revision surgery instead of IOP at last follow-up was taken into account (i.e., IOP either prior to the first revision surgery or at last follow-up in case of absence of revision surgeries, with or without AGM), the mean IOP was 19.9 ± 9.4 mmHg after modified canaloplasty versus 13.3 ± 6.3 mmHg after trabeculectomy (*p* < 0.001). Figure 2B displays IOP either at the last follow-up (bright brown for canaloplasty, bright blue for trabeculectomy) or prior to revision surgery (dark brown for canaloplasty, dark blue for trabeculectomy), showing a high number of canaloplasty cases above the 18 mmHg and 20% IOP reduction threshold lines.

### 3.3. Antiglaucomatous Medications (AGM) and Revision Surgery

During the follow-up time, 35/70 (50.0%) of modified canaloplasty subjects needed AGM compared to 15/140 (10.7%) in the trabeculectomy group (*p* < 0.001). After modified canaloplasty, the number of AGM was reduced from 2.8 ± 1.1 to 0.8 ± 1.0, whereas the number of AGM in the trabeculectomy group was 3.2 ± 1.0 preoperatively and 0.1 ± 0.3 postoperatively.

Revision surgery was necessary in 29/70 (41.4%) of modified canaloplasty cases and in 30/140 (21.4%) of trabeculectomy cases (*p* = 0.004). The median time to first revision surgery was 0.58 [IQR 0.18; 2.28] years for canaloplasty cases and 2.03 [IQR 0.81; 3.92] years for trabeculectomy cases. Table 3 shows the performed first revision surgeries.

In the case of circumferential suture trabeculotomy, the canaloplasty prolene suture was grasped ab interno with 25-gauge forceps and pulled over 360° to open up Schlemm’s canal.

### 3.4. Surgical Complications

Surgical complications occurred in 10/70 (14.5%) of eyes after modified canaloplasty and in 10/134 (7.5%) after trabeculectomy (*p* = 0.19). The most common complication after modified canaloplasty was hyphema (4/10, 40%) followed by hypotony (2/10, 20%), choroidal effusion (1/10, 10%), IOP decompensation (1/9, 10%), iris incarceration (1/10, 10%), and synechiae (1/10, 10%). After trabeculectomy, the most common complication was controlled hypotony (4/10, 40%), followed by IOP decompensation (2/10, 20%), scleral thinning (2/10, 20%), choroidal effusion (1/10, 10%), and malignant glaucoma (1/10, 10%).

### 3.5. Visual Acuity

Preoperative visual acuity (VA) was 0.24 ± 0.4 LogMAR in the modified canaloplasty group and 0.41 ± 0.4 LogMAR in the trabeculectomy group (*p* = 0.003). At the last visit, the VA amounted to 0.40 ± 0.6 LogMAR versus 0.39 ± 0.32 LogMAR (*p* = 0.77). The mean difference between preoperative VA and postoperative IOP was −0.17 LogMAR (*p* = 0.01) in the modified canaloplasty group and 0.03 (*p* = 0.26) in the trabeculectomy group (paired *t*-test).

### 3.6. Subgroup Analysis: Results of POAG Cases

**Figure 3:**
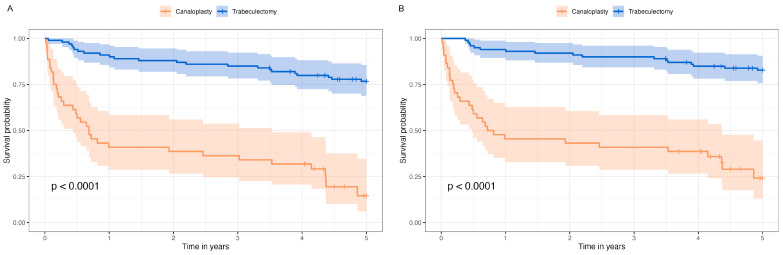
Kaplan–Meier survival analysis of complete surgical success (**A**) and qualified surgical success (**B**) after modified canaloplasty and trabeculectomy in primary open-angle glaucoma cases (POAG).

Figure 3A shows the survival probability of complete surgical success and Figure 3B the survival probability of qualified surgical success in Kaplan–Meier survival analysis for POAG cases. Cox regression analysis of qualified success revealed an HR of 7.6 (*p* < 0.001) for cases after modified canaloplasty, independent of age and sex. The median survival probability was 0.77 [95%CI 0.48, 4.37]. Details on success, IOP, AGM, revision surgeries, complications, and visual acuity are presented in Table 4.

## 4. Discussion

The aim of this study was to evaluate the long-term success, efficacy, and safety of modified canaloplasty versus trabeculectomy with a minimum follow-up time of 3 years. Although the trabeculectomy group had a longer mean follow-up time, it showed a better survival probability, lower IOP at follow-up, and less need for AGM or revision surgeries, whereas the occurrence of surgical complications was not statistically significantly higher after trabeculectomy.

Survival analysis demonstrated the inferiority of modified canaloplasty compared to trabeculectomy: 50% of canaloplasty cases had failed after 12 months when applying qualified success criteria (IOP between 5 and 18 mmHg under AGM if necessary, no revision surgery). The risk of failure was more than four times higher during the first year after surgery than afterward. Surgical success after the follow-up period was more than twice as high after trabeculectomy than after modified canaloplasty, independently from how success was defined. Previous studies showed inconsistent findings regarding surgical success: Some studies found similar results to this study with a higher complete success after trabeculectomy [12,16,17,18,19], while in other studies both methods reached comparable results [20,21]. A long-termed follow-up period of up to three years in a mixed cohort was reported by Nassri et al. [12] and there was a similar gap of complete success between canaloplasty and trabeculectomy as in the present study. A prospective randomized controlled study by Matlach et al. found 2-year complete success rates (IOP threshold ≤ 18 mmHg) of roughly 40% after canaloplasty, which is similar to the 2-year canaloplasty complete success rates in this study [16]. Liu et al. reported higher success after trabeculectomy in a meta-analysis [19]. In contrast, two other meta-analyses did not reveal a statistically significant difference in strict, complete, or qualified success between both methods, however, the success analyses were restricted to two to three publications with small case numbers [22,23]. The varying results may be explained by the different definitions of success rates among the previous studies impeding comparability.

Trabeculectomy was more efficient in terms of IOP control with a lower IOP at follow-up than after modified Canaloplasty and a significantly better absolute and percentage IOP decrease which was similarly described in previous studies and meta-analyses [10,18,19,20,22,23,24,25]. Brüggemann et al., Matlach et al., and Nassri et al. reported non-significant differences in IOP between the two groups after 12 months or beyond; however, IOP was constantly lower in the trabeculectomy groups [12,16,21]. The superiority of trabeculectomy was also present after restricting the analysis to cases without AGM and revision surgeries.

Throughout the follow-up period, every second modified canaloplasty case needed supplemental AGM, whereas 89.3% of trabeculectomy cases did not, and the number of medications was significantly lower after trabeculectomy than after modified canaloplasty. The same trend (either significant or not) was visible in all previous comparative studies [12,16,17,18,20,22,23,24,25,26] except the study of Brüggemann et al., where none of the canaloplasty cases and three of the trabeculectomy cases were on topical AGM [21]. The meta-analysis by Zhang et al. found that AGM was slightly more reduced after trabeculectomy than after canaloplasty by 0.37 medicaments (*p* = 0.11) [23]; the two other meta-analyses by Liu et al. and Rulli et al., however, they did not analyze the number or reduction of AGM [10,19].

Non-penetrating procedures have been developed to avoid surgical complications which are feared to occur in high proportions after trabeculectomy. The decision for trabeculectomy or non-penetrating surgery mainly depends on the individual risk for adverse events, e.g., high myopia or non-compliance with the postoperative therapy regimen. Adverse events range from transient hypotony over permanent hypotony with choroidal detachment and/or hemorrhage to bleb leak, blebitis, and vision-threatening endophthalmitis. The prospective Collaborative Bleb-related Infection Incidence and Treatment Study (CBIITS) found a 5-year cumulative incidence of bleb-related endophthalmitis of 2.2 ± 0.5% after MMC-augmented trabeculectomy [27]. Contrarily, no reports of endophthalmitis following canaloplasty have been reported yet, however, the presence of a filtering bleb carrying the danger for blebitis has been described in up to 10% of cases after canaloplasty [28]. Compared to canaloplasty, a higher occurrence of postoperative hypotony after trabeculectomy has been reported with a proportion of 16–37% [20,26]. Zhang et al. found 9-times higher odds for hyphema (*p* < 0.001) in the canaloplasty groups, whereas the odds were a third (*p* = 0.01) for hypotony and a quarter (*p* = 0.03) for choroidal effusion/detachment in the canaloplasty groups compared to trabeculectomy groups. Although canaloplasty is not a penetrating procedure, hyphema is likely to occur because of blood reflux from the collector channels, indicating a functioning outflow pathway and the correct position of the suture [29]. The results of the present study are in concordance with the previous results: The most common complication after canaloplasty was hyphema, whereas hypotony prevailed after trabeculectomy. Overall, the number of complications was similar in both groups. Visual acuity decreased significantly from 0.24 ± 0.4 LogMAR preoperatively to 0.40 ± 0.6 LogMAR at the end of follow-up after modified canaloplasty (*p* = 0.01), while it was stable after trabeculectomy (0.41 ± 0.4 LogMAR preoperatively to 0.39 ± 0.32 LogMAR at the end of follow up, *p* = 0.26). A possible explanation might be the almost two times higher revision rate after canaloplasty compared to trabeculectomy.

Trabeculectomy showed superior long-term results than canaloplasty in this study, yet there still might be reasonable indications for canaloplasty in the clinical setting. In highly myopic eyes, postsurgical complications, in particular hypotony with subsequent choroidal detachment or hemorrhage, are considered to occur more frequently due to the thin sclera with low rigidity being more likely to collapse [30]. Although two previous studies did not show a higher risk for failure or complications after trabeculectomy in myopic patients [25,31], canaloplasty might be a safer option since there is no absolute hypotony phase due to the intact Descemet’s membrane.

Furthermore, trabeculectomy needs more intensive postsurgical care related to the risk of bleb scarring [32]. Frequent visits and the application of subconjunctival anti-fibrotic 5-fluorouracil are necessary to ensure a successful filtering effect. Eye rubbing or unhygienic handling of a recent trabeculectomy can cause serious vision-threatening complications. Some patients might not comply with these requirements and thus may achieve a better result by receiving a canaloplasty.

Lastly, canaloplasty in combination with antiglaucomatous medication can achieve a satisfying IOP stabilization and therefore might be an adequate therapy. Surgeons should inform their patients about the expected IOP decreasing effect and rates for postoperative AGM and revision surgeries and make an individual, shared decision.

Several limitations must be mentioned: First, the distribution of diagnosis differed between the groups with a higher proportion of secondary glaucoma in the modified canaloplasty group. As IOP sometimes is more difficult to control in secondary glaucoma, the success of modified canaloplasty might be falsely low. Second, 30 patients of the canaloplasty group were lost to follow-up and excluded prior to the study (not included in the analysis). Due to the multilevel healthcare system in Germany, follow-up examinations are usually performed by the general ophthalmologist. Patients are only referred to the clinic in case of difficulties such as a re-increase of IOP. Thus, a high proportion of the lost patients might have had a successful surgery, which did not require further referrals, leading to falsely high failure rates. Failure in canaloplasty often directly led to revision surgery while there are also less invasive revision procedures, such es YAG-goniopuncture, that may be rather comparable to suture lysis in case of trabeculectomy. Third, the proportional hazard assumption was not met for the Cox regression analysis, which might be caused by the high number of events during the first year. Although the graphical presentation of Schoenfeld residuals did not indicate non-proportional hazards, the hazard ratio thus should be carefully interpreted.

## 5. Conclusions

This retrospective analysis showed the superiority of trabeculectomy in terms of survival probability, IOP decrease, and the necessity for anti-glaucomatous medication and revision surgery compared to modified canaloplasty after a median follow-up period of 4.6 and 5.8 years in open-angle glaucoma patients, whereas the occurrence of complications was similar in both groups.

## Figures and Tables

**Table 1:** Baseline characteristics.

	Modified Canaloplasty (n = 70)	Trabeculectomy (n = 140)
Age [years]	60.0 ± 13.8	63.0 ± 12.2
Sex, female	43 (61.4%)	81 (57.9%)
Laterality, OD	35 (50.0%)	67 (47.9%)
Diagnosis		
POAG	44 (62.9%)	100 (71.9%)
NTG	4 (5.7%)	14 (10.1%)
Secondary OAG	22 (31.4%)	25 (18.0%)
PXFG	7 (10.0%)	15 (10.7%)
PG	8 (11.4%)	2 (1.4%)
following uveitis	3 (4.3%)	3 (2.1%)
following contusion	1 (1.4%)	1 (0.7%)
following surgery	3 (4.3%)	1 (0.7%)
Angle abnormality	0 (0.0%)	3 (2.1%)
Maximum IOP [mmHg]	35.3 ± 11.5	31.3 ± 10.5
Preoperative IOP [mmHg]	24.0 ± 6.9	25.3 ± 9.9
Preoperative number of AGM	2.8 ± 1.1	3.2 ± 1.0
Preoperative MD of visual fields [dB]	6.6 [IQR 3.2, 13.4]	7.0 [IQR 3.8, 13.9]
- range	0.1 to 23.1	0.3 to 27.6
Visual acuity [LogMAR]	0.24 ± 0.4	0.41 ± 0.4
Duration of glaucoma [years]	8.2 [IQR 2.6, 13.4]	7.6 [IQR 3.4, 14.8]
Follow-up time [years]	4.6 [IQR 4.3, 5.05]	5.8 [IQR 5.4, 6.3]

Abbreviations: OD: Oculus dexter, POAG: Primary Open-Angle Glaucoma, NTG: Normal Tension Glaucoma, OAG: Open-Angle Glaucoma, IOP: Intraocular Pressure, AGM: Antiglaucomatous Medications, MD: Mean defect, PXFG: Pseudoexfolative glaucoma, PG: Pigmentary glaucoma.

**Table 2:** Multivariable Cox regression (for qualified success).

	Hazard Ratio	95%-CI	*p* Value
Patient age [years]	1.01	0.99, 1.03	0.33
Sex			
male	(Reference)		
female	1.29	0.77, 0.78	0.32
Diagnosis			
POAG	(Reference)		
NTG	0.83	0.35, 1.96	0.67
Secondary OAG	1.04	0.97, 0.60	0.90
Surgery			
Trabeculectomy	(Reference)		
**Canaloplasty**	**6.03**	**3.66, 9.93**	**<0.001**

POAG: Primary Open-Angle Glaucoma, NTG: Normal Tension Glaucoma, OAG: Open-Angle Glaucoma, 95%-CI: 95%-Confidence interval.

**Table 3:** First revision surgeries after modified canaloplasty and trabeculectomy.

	Modified Canaloplasty	Trabeculectomy
Number of patients with revision surgery	29 (41.4%)	30 (21.4)
Needling	3 (4.3%)	20 (14.3%)
Trabeculectomy	19 (27.1%)	0 (0.0%)
Transconjunctival flap resuturing	1 (1.4%)	5 (3.6%)
Circumferential suture trabeculotomy	3 (4.3%)	0 (0.0%)
Cyclophotocoagulation	2 (2.9%)	0 (0.0%)
Cyclocryotherapy	0 (0.0%)	1 (0.7%)
Bleb revision	1 (1.4%)	2 (1.4%)
iStent implantation	0 (0.0%)	1 (0.7%)

**Table 4:** Subgroup analysis of cases with primary open-angle glaucoma.

	Modified Canaloplasty (n = 44)	Trabeculectomy (n = 100)	*p* Value
Age [years]	61.5 ± 12.5	63.0 ± 11.1	0.54
Sex	29 (65.9%)	59 (59.9%)	0.55
Strict success	6 (13.6%)	60 (60.0%)	<0.001
Complete success	9 (20.5%)	71 (71.0%)	<0.001
Qualified success	13 (29.5%)	76 (76.0%)	<0.001
Preoperative IOP [mmHg]	23.7 ± 7.1	28.7 ± 10.3	0.25
Postoperative IOP [mmHg]	14.8 ± 4.2	11.8 ± 4.3	<0.001
IOP decrease absolute [mmHg] (relative)	2.5 ± 7.5 (32.0 ± 25.0%)	14.0 ± 10.7 (48.8 ± 24.8%)	0.003 (<0.001)
Preoperative AGM	2.7 ± 1.1	3.2 ± 1.0	0.007
Postoperative AGM	0.9 ± 1.0	0.09 ± 0.3	0.001
Revision surgery	19 (43.2%)	19 (19.0%)	0.005
Complications	5 (11.6%)	7 (7.5%)	0.65
Preoperative VA [LogMAR]	0.27 ± 0.45	0.39 ± 0.35	0.10
Postoperative VA [LogMAR]	0.37 ± 0.57	0.37 ± 0.30	0.98

Abbreviations: IOP: Intraocular pressure, AGM: Antiglaucomatous medication, VA: Visual acuity.

## Data Availability

The data presented in this study are available on request from the corresponding author. The data are not publicly available due to their containing information that could compromise the privacy of research participants.

## Supplementary Materials

The following supporting information can be downloaded at: https://www.mdpi.com/article/10.3390/life13020516/s1, Supplemental Material File S1: Cox regression analysis with additional variables.
