# Supplementary material for: Long-Term Efficacy and Safety of Modified Canaloplasty Versus Trabeculectomy in Open-Angle Glaucoma
Source: Life (Basel). 2023 Feb 13;13(2):516. doi: 10.3390/life13020516 (PMC9963969; doi:10.3390/life13020516)
Supplement: Supplementary file 1 [file life-13-00516-s001.zip › life-2111719-supplementary.pdf]

**Supplemental material 1: Cox regression analysis with additional variables**

Cox regression analysis for complete success

| | HR | lower .95 | upper .95 | p value |
|---|---|---|---|---|
| Age [years] | 1.011841 | 0.9914 | 1.033 | 0.258 |
| Sex (female) | 1.315294 | 0.7954 | 2.175 | 0.285 |
| Diagnose (NTG) | 1.078282 | 0.5208 | 2.233 | 0.839 |
| Diagnose (Secondary) | 1.393801 | 0.7831 | 2.481 | 0.259 |
| Method (Canaloplasty) | 5.031579 | 3.0564 | 8.283 | $2.12 \times 10^{-10}$ *** |
| Preoperative MD [dB] | 1.003420 | 0.9675 | 1.041 | 0.854 |
| Duration glaucoma [years] | 0.995620 | 0.9705 | 1.021 | 0.736 |

---
Signif. codes:  0 ‘***’ 0.001 ‘**’ 0.01 ‘*’ 0.05 ‘.’ 0.1 ‘ ’ 1

Cox regression analysis for qualified success

| | HR | lower .95 | upper .95 | p |
|---|---|---|---|---|
| Age [years] | 1.0155 | 0.9948 | 1.037 | 0.1439 |
| Sex (female) | 1.6629 | 0.9494 | 2.913 | 0.0753 |
| Diagnose (NTG) | 0.9902 | 0.4397 | 2.230 | 0.981 |
| Diagnose (Secondary) | 1.0638 | 0.5728 | 1.976 | 0.8447 |
| Method (Canaloplasty) | 6.2464 | 3.5443 | 11.009 | $2.35 \times 10^{-10}$ *** |
| Preoperative MD [dB] | 0.9782 | 0.9404 | 1.018 | 0.2747 |
| Duration glaucoma [years] | 0.9984 | 0.9711 | 1.027 | 0.9124 |

---
Signif. codes:  0 ‘***’ 0.001 ‘**’ 0.01 ‘*’ 0.05 ‘.’ 0.1 ‘ ’ 1
